# Genome-wide association of changes in swine feeding behaviour due to heat stress

**DOI:** 10.1186/s12711-018-0382-1

**Published:** 2018-03-25

**Authors:** Amanda J. Cross, Brittney N. Keel, Tami M. Brown-Brandl, Joseph P. Cassady, Gary A. Rohrer

**Affiliations:** 1 Department of Animal Science, South Dakota State University, Brookings, SD 57007 USA; 2 USDA, ARS, U.S. Meat Animal Research Center, Clay Center, NE 68933 USA

## Abstract

**Background:**

Heat stress has a negative impact on pork production, particularly during the grow-finish phase. As temperature increases, feeding behaviour changes in order for pigs to decrease heat production. The objective of this study was to identify genetic markers associated with changes in feeding behaviour due to heat stress. Feeding data were collected on 1154 grow-finish pigs using an electronic feeding system from July 2011 to March 2016. In this study, days were classified based on the maximum temperature humidity index (THI) during the day as “Normal” (< 23.33 °C), “Alert” (23.33 °C ≤ × < 26.11 °C), “Danger” (26.11 °C ≤ × < 28.88 °C), and “Emergency” (≥ 28.88 °C). Six hundred and eighty-one pigs that experienced more than one THI category were genotyped using a variety of SNP platforms, with final genotypes imputed to approximately 60,000 markers.

**Results:**

A genome-wide association study (GWAS) for change in feeding behaviour between each pair of THI categories (six pairs) was conducted. Estimates of heritability for differences in feeding activity between each of the THI categories were low (0.02 ± 0.03) to moderate (0.21 ± 0.04). Sixty-six associations which explained more than 1% of the genomic variation for a trait were detected across the six GWAS, with the smallest number of associations detected in comparisons with Emergency THI. Gene ontology enrichment analysis showed that biological processes related to immune response and function were over-represented among the genes located in these regions.

**Conclusions:**

Genetic differences exist for changes in feeding behaviour induced by elevated ambient temperatures in grow-finish pigs. Selection for heat-tolerant grow-finish pigs should improve production efficiency during warm months in commercial production. Genetic variation in heat shock, stress response and immune function genes may be responsible for the observed differences in performance during heat stress events.

## Background

Heat stress is a major economic concern in the swine industry. In the USA, economic losses due to heat stress are estimated at $300 million per year, of which a majority occur during the grow-finish phase [1]. Production losses due to heat stress result from decreased growth of market hogs, reduced feed intake, and mortality [2–4].

Swine feeding behavioural patterns change as temperature increases. Pigs spend less time eating and more time lying down during high temperatures [5, 6] and change eating behaviour, mealtime, and meal size [5, 7]. Nienaber et al. [8] showed that reducing meal size and the number of meals per day can reduce the effects of high temperatures on heat production by decreasing physical and metabolic activity.

Although there have been several advances in production management and barn cooling systems, production efficiency continues to suffer during warm months. Pigs have a thermal comfort zone in which they are most productive, which depends on several factors, including sex, genetics, relative humidity, and velocity of ambient air [9, 10].

Genetic selection for increased growth is associated with a decrease in a pig’s ability to handle heat stress [11]. Thus, genetic markers that are associated with heat stress could be used to select for and breed more heat-resilient pigs. The objective of this study was to identify genetic markers associated with changes in feeding behaviour due to heat stress in grow-finish pigs.

## Methods

All animal protocols conformed to procedures outlined in the *Guide for care and use of agricultural animals in agricultural research and teaching* [12] and were approved by the USMARC Institutional Animal Care and Use Committee.

### Phenotypic data collection

Phenotypic data were collected on grow-finish pigs (n = 1648), which were reared at the U.S. Meat Animal Research Center from July 2011 to March 2016. Pigs were placed in a barn in grow-finish groups (n = 7) of approximately 240 pigs at 8 to 10 weeks of age. Barrows and gilts were mixed and distributed into six pens, with 39 to 40 pigs per pen. Three sire lines, Duroc, Landrace, and Yorkshire, were represented and all dams were from a Landrace–Yorkshire composite population. On average, within a grow-finish group there were 6.2 full-sibs and 25.9 paternal half-sibs represented. Animals were tagged with a low-frequency electronic identification tag upon entry into the grow-finish barn.

Pens were fitted with an electronic feeding system that monitored feeding behaviour, as described by Brown-Brandl et al. [13]. Briefly, each pen had one feeder with five slots, allowing up to five animals to eat at any given time. Pigs were provided ad libitum access to a corn-soybean meal diet that was designed to meet or exceed an animal’s nutrient requirements. Each feeder slot was fitted with an antenna and a multiplexer. Every 20 s, the device determined which pigs are located at the feeder and then recorded animal number, feeder position and time, which will be referred to as ‘RFID pings’ hereafter. Data were collected over a 4-month period for each group of pigs.

Each hour, the temperature humidity index (THI) was calculated [14] using outside temperature (°C) and relative humidity (RH) as:

$${\text{THI}}\left( {^\circ {\text{C}}} \right) \, = {\text{ T}}\left( {^\circ {\text{C}}} \right) \, {-} \, \left[ {0.55 \, {-} \, \left( {0.0055 \, \times {\text{ RH}}} \right)} \right] \, \times \, \left[ {{\text{T}}\left( {^\circ {\text{C}}} \right) \, {-} \, 14.5} \right]$$

Days were classified into THI categories based on the maximum THI, as outlined by Brown-Brandl et al. [15]. THI categories included “Normal” (< 23.33 °C), “Alert” (23.33 °C ≤ × < 26.11 °C), “Danger” (26.11 °C ≤ × < 28.88 °C), and “Emergency” (≥ 28.88 °C). It should be noted that not all animals experienced every THI category. Only 949 animals experienced a THI greater than Normal. For each animal, the total number of RFID pings was computed for each day, and the average number of RFID pings per day was computed for each THI category. Similarly, the average number of RFID pings per day was computed for each breed by sex combination for each THI category. The difference between an animal’s average number of RFID pings in a specific THI category and the corresponding breed-sex mean was computed and standardized to a mean of 0 and a standard deviation of 1 for each THI category. Differences in feeding behaviour between two THI categories (e.g. Alert-Normal) were quantified by calculating the difference in standardized RFID pings between the two categories. Therefore, if an animal experienced all four THI categories during the finishing phase, four standardized THI feeding behaviour values were computed, which reflected how this animal’s behaviour deviated from that of a typical animal of this breed type and sex, and six values were computed that indicated how it responded to different temperatures relative to its breed type-sex contemporaries. Not all animals experienced all four THI categories during grow-finish and loss of electronic tags resulted in varying numbers of animals with data for each comparison. It was assumed that animals that reduced their feeding activity more than their breed type-sex contemporaries as the THI category increased, were more affected by heat stress. Phenotypic correlations among the six traits analysed are in Table 1.

**Table 1:** Phenotypic correlations among the six temperature-humidity index (THI) category comparisons analysed

THI comparison	Normal-Danger	Normal-Emergency	Alert-Danger	Alert-Emergency	Danger-Emergency
Normal-Alert	0.876	0.684	0.245	0.248	0.180
Normal-Danger		0.832	0.682	0.506	0.326
Normal-Emergency			0.675	0.874	0.795
Alert-Danger				0.704	0.420
Alert-Emergency					0.939

Normal (× < 23.33 °C), Alert (23.33 °C ≤ × < 26.11 °C), Danger (26.11 °C ≤ × < 28.88 °C), and Emergency (× ≥ 28.88 °C)

### Genotypic data

Tail samples were collected on all pigs and stored at − 20 °C. Genomic DNA was extracted using the WIZARD genomic DNA purification kit according to the manufacturer’s protocol (Promega Corp., Madison, WI, USA). Genotyping was conducted using three platforms: the NeoGen Porcine GGPHD chip (GeneSeek, Lansing, USA), the Illumina Porcine SNP60 V2 chip (Illumina, Inc., San Diego, USA), and the NeoGen GGP-Porcine chip (GeneSeek, Lansing, USA). Quality control involved filtering out genotypes that had a minor allele frequency lower than 5% and that did not have a unique map position in the Sscrofa10.2 genome assembly [16]. After quality control, 58,096 single nucleotide polymorphisms (SNPs) from the GGPHD chip, 38,598 SNPs from the Porcine SNP60 V2 chip, and 6882 SNPs from the GGP-Porcine chip were retained for use in subsequent analyses. In total, 1118 pigs were genotyped using the GGPHD chip, two pigs were genotyped using the SNP60 V2 chip, and 34 pigs were genotyped using the GGP-Porcine chip. Genotypes for animals genotyped on the Porcine SNP60 V2 chip and GGP-Porcine chip were imputed to the NeoGen Porcine GGPHD chip (number of SNPs = 58,096) by pedigree imputation using FImpute v2.2 [17].

### Genome-wide association study (GWAS)

Each of the six traits (difference between the standardized feeding behaviour of two THI categories) was analysed using a mixed linear model with sex, sire breed, and contemporary group as fixed effects. Contemporary group was the combined effect of farrowing group and pen. Two farrowing groups (year–week of birth) were represented in each grow-finish group and the barn contained six pens. Although phenotypes were deviations from the animal’s sex and breed of sire means, breed of sire and sex were included as fixed effects to account for population stratification that may be present in the genotypic data. Genomic regions associated with each trait were identified and quantified using a Bayes-C variable selection method and GenSel software [18] based on the following modified statistical model [18]:

$${\mathbf{y}} = {\mathbf{X}}{\varvec{\upbeta}} + {\mathbf{Zu}} + {\mathbf{e}},$$

where ${\mathbf{y}}$ is a vector of trait phenotypes (differences in feeding behavior between two THI categories), ${\mathbf{X}}$ is an incidence matrix of fixed effects (${\varvec{\upbeta}}$), ${\mathbf{Z}}$ is a matrix of SNP genotypes with non-zero effects (proportion determined as 1 − π) that were fitted as random effects (${\mathbf{u}}$) distributed N (0, $\sigma_{u}^{2}$), and ${\mathbf{e}}$ is a vector of random residual effects assumed to be normally distributed N (0, $\sigma_{{\mathbf{e}}}^{2}$).

Priors for genetic and residual variances and the prior proportion of SNPs that are assumed to have no effect on the trait within an iteration of the Monte Carlo Markov chain (MCMC) (π) for each trait were obtained by running Bayes-Cπ using GenSel [18] with the same model as described above. Priors used for Bayes-Cπ analyses were the same for all THI category comparisons and were 0.98, 0.10 and 0.10 for π, genetic variance and residual variance, respectively. These analyses were run for a minimum of 8100 iterations, with the first 100 discarded as burn-in. Plots of π over iterations were evaluated to determine if additional iterations were necessary to obtain a converged estimate. Resulting values for π used in Bayes-C analyses are in Table 2.

**Table 2:** Posterior estimates of π values obtained from Bayes-Cπ analyses and used in Bayes-C analyses for each of the temperature-humidity index (THI) category comparisons

THI category comparison^a^	π
Normal-Alert	0.999893
Normal-Danger	0.999815
Normal-Emergency	0.999906
Alert-Danger	0.999954
Alert-Emergency	0.999947
Danger-Emergency	0.999943

^a^Normal (× < 23.33 °C), Alert (23.33 °C ≤ × < 26.11 °C), Danger (26.11 °C ≤ × < 28.88 °C), and Emergency (× ≥ 28.88 °C)

For the Bayes-C analyses, a chain of 41,000 iterations was used, with the first 1000 cycles discarded as burn-in. Effects were sampled every 40 iterations to obtain a posterior distribution for the genetic variance. Genomic regions associated with each trait were identified using 1-Mb genome windows following Wolc et al. [19]. The standard deviation of marker-based estimates of heritability was calculated as the standard deviation of the heritability estimates of the last 100 samples.

### Functions of genes in significant genomic regions

Genes located in 1-Mb windows explaining more than 1.0% of the genomic variance were obtained using the NCBI annotation of Sscrofa10.2 (Release 104). Two gene lists were analysed. The first contained genes located in all 1-Mb windows that were detected in the six traits. The second list only included genes located in 1-Mb windows that explained more than 3.0% of the genomic variance for at least one trait. For the latter list, if two adjacent windows exceeded 3.0% of the genomic variance, then only the 1-Mb region with the greatest estimated effect was included in the analysis.

The PANTHER classification system (version 12.0; http://www.pantherdb.org/) [20] was used to determine the functions of genes in these lists. Enrichment analysis of gene function was performed using PANTHER’s implementation of the binomial test of overrepresentation [20], which determines whether the list of genes contains more genes involved in a particular pathway or function than would be expected at random at a Bonferroni corrected *p* value less than 0.05. Significance of gene ontology (GO) terms was assessed using the default Ensembl *Sus scrofa* GO annotation as background for the enrichment analysis.

## Results

### Feeding behaviour patterns by breed and sex

Of the 1648 grow-finish pigs (727 barrows and 921 gilts) analyzed in this study, 309 were Duroc sired, 786 were Landrace sired, and 553 were Yorkshire sired. In all three sire breeds, feeding activity of barrows, as determined by the average number of RFID pings per day, exceeded that of gilts for all THI categories (Table 3). Yorkshire and Duroc sired pigs had greater feeding activity than Landrace sired pigs across all THI categories (Table 3). For Yorkshire and Duroc sired pigs, feeding activity increased as THI increased, while the opposite trend was observed for Landrace sired pigs.

**Table 3:** Average number of RFID pings per day (mean ± standard error) by sire breed and sire breed-sex for each temperature-humidity index (THI) category

Breed	Sex	Normal × < 23.33 °C	Alert 23.33 ≤ × < 26.11 °C	Danger 26.11 ≤ × < 28.88 °C	Emergency × ≥ 28.88 °C
*Duroc*					
	All	153.4 ± 0.5	168.9 ± 0.9	172.7 ± 1.1	182.6 ± 3.0
	Number	309	156	155	152
	Barrow	154.8 ± 0.7	179.0 ± 1.4	183.5 ± 1.7	194.3 ± 4.4
	Number	155	78	78	75
	Gilt	152.0 ± 0.6	159.1 ± 1.2	162.1 ± 1.4	171.1 ± 4.0
	Number	154	78	77	77
*Yorkshire*					
	All	140.9 ± 0.4	145.3 ± 0.7	150.0 ± 0.9	157.8 ± 3.1
	Number	553	397	387	256
	Barrow	156.5 ± 0.7	160.5 ± 1.3	170.7 ± 1.6	188.4 ± 4.7
	Number	214	138	137	84
	Gilt	130.9 ± 0.5	135.5 ± 0.8	137.5 ± 1.0	137.2 ± 3.6
	Number	339	259	250	172
*Landrace*					
	All	134.3 ± 0.3	122.5 ± 0.6	108.5 ± 0.7	65.8 ± 1.0
	Number	786	392	396	390
	Barrow	140.3 ± 0.4	131.7 ± 0.9	118.0 ± 1.1	71.2 ± 1.7
	Number	358	160	161	159
	Gilt	129.3 ± 0.3	115.6 ± 0.7	101.7 ± 0.8	62.4 ± 1.1
	Number	428	232	235	231

### GWAS

Estimates of heritability from the Bayes-C analyses of GenSel for each THI category comparison are in Table 4 and details for each 1-Mb window that explained more than 1% of the genomic variance are in Table 5. Changes in behaviour between Normal and Alert THI categories showed a modest heritability of 0.14 (± 0.04), with more than 58% of the genomic variance explained by 17 regions on nine chromosomes. Five regions on *Sus scrofa* (SSC) chromosome 5 accounted for 24.9% of genomic variance and SSC13 had four regions that accounted for 8.9% of the genomic variance. SSC7 and 17 each had two regions that jointly accounted for 6.0 and 7.4% of the genomic variance, respectively.

**Table 4:** Posterior estimates of heritability for changes in feeding behaviour for each temperature-humidity index (THI) category comparison

THI category comparison^a^	Number of animals	Range of values	Genomic variance	Residual variance	Heritability (SD)^b^
Normal-Alert	681	− 2.07 to 1.85	0.0249	0.1577	0.136 (0.044)
Normal-Danger	681	− 2.91 to 2.90	0.0602	0.2326	0.205 (0.044)
Normal-Emergency	561	− 3.84 to 2.42	0.0338	0.4028	0.077 (0.045)
Alert-Danger	681	− 1.94 to 2.48	0.0050	0.0700	0.070 (0.031)
Alert-Emergency	561	− 3.12 to 1.74	0.0085	0.2303	0.036 (0.028)
Danger-Emergency	561	− 2.72 to 1.19	0.0034	0.1708	0.020 (0.026)

^a^Normal (× < 23.33 °C), Alert (23.33 °C ≤ × < 26.11 °C), Danger (26.11 °C ≤ × < 28.88 °C), and Emergency (× ≥ 28.88 °C)

^b^*SD* standard deviation of the last 100 samplings of the GenSel BayesC analysis

**Table 5:** One-Mb windows that explained more than 1% of the genetic variance for each temperature-humidity index (THI) category comparisons

THI category comparison^a^	Chromosome	Position^b^ (Mb)	% of genetic variance explained	Number of SNPs
*Normal-Alert*				
	5	68	11.9	26
	10	44	6.2	24
	5	108	5.6	28
	7	53	4.8	28
	17	2	4.8	32
	5	107	4.5	31
	13	85	3.1	7
	6	15	2.8	39
	13	17	2.8	30
	17	3	2.6	29
	5	109	1.8	30
	13	16	1.8	27
	X	3	1.3	63
	13	84	1.2	14
	7	44	1.2	22
	5	106	1.1	23
	15	140	1.0	60
*Normal-Danger*				
	7	53	13.2	28
	10	44	12.7	24
	1	228	2.6	13
	1	22	2.6	29
	1	224	1.8	12
	5	109	1.8	30
	13	77	1.5	13
	6	15	1.5	39
	7	134	1.2	32
	5	61	1.2	21
	7	45	1.0	26
	7	52	1.0	28
	6	77	1.0	21
*Normal-Emergency*				
	14	11	8.3	42
	12	12	5.2	35
	13	17	2.7	30
	17	61	2.5	50
	12	13	2.0	39
	X	3	1.8	
	14	13	1.8	27
	X	2	1.4	44
	2	10	1.3	38
	5	109	1.2	30
	18	39	1.1	15
	13	203	1.1	54
	10	32	1.1	20
*Alert-Danger*				
	1	228	19.0	13
	1	226	14.3	13
	7	53	10.2	28
	7	52	6.2	28
	7	134	5.0	32
	1	227	4.4	10
	1	224	3.1	12
	17	22	2.3	20
	15	30	2.1	20
	1	283	1.5	29
	1	223	1.4	7
	12	12	1.4	35
	17	4	1.0	32
*Alert-Emergency*				
	14	11	9.8	42
	14	13	7.5	27
	1	15	1.5	49
	12	12	1.4	35
	14	10	1.4	41
	15	30	1.4	20
*Danger-Emergency*				
	14	11	4.2	42
	1	15	1.4	49
	15	117	1.1	15
	4	77	1.0	28

^a^Normal (× < 23.33 °C), Alert (23.33 °C ≤ × < 26.11 °C), Danger (26.11 °C ≤ × < 28.88 °C), and Emergency (× ≥ 28.88 °C)

^b^Positions are based on build 10.2 of the swine genome

Feeding behaviour changes between Normal and Danger THI had the highest estimate of heritability (0.21 ± 0.04). Over 40% of the genomic variance was explained by 13 regions on six chromosomes. Four regions on SSC7 jointly accounted for 16.5% of the genomic variance, while SSC1 had three regions that jointly accounted for 7.1% of the genomic variance. The estimate of heritability for the Normal-Emergency comparison was considerably lower (0.08 ± 0.04), with approximately 30% of genomic variance explained by 13 regions on nine chromosomes. Regions on SSC14 and 12 accounted for the highest percentage of genomic variance (10.1 and 7.3%, respectively).

The estimate of heritability for changes in feeding behaviour between the Alert and Danger categories was similar to that for the Normal-Emergency categories (0.07 ± 0.03) and 72% of the genomic variance was explained by regions on five chromosomes. SSC1 had six regions that jointly explained 43.8% of the genomic variance and SSC7 had three regions that jointly explained 21.5% of the genomic variance. The estimate of heritability for the Alert-Emergency THI comparison was 0.04 ± 0.03 and 23% of the genomic variance was explained by six regions on four chromosomes. SSC14 had three regions that jointly accounted for 18.7% of the genomic variance. Almost 8% of the genomic variance was explained by four regions on separate chromosomes for the Danger-Emergency THI comparison. SSC14 explained the largest proportion of genomic variance (4.2%). The estimate of heritability for this comparison (0.02 ± 0.03) was the lowest of the six THI comparisons.

### Functions of genes in significant regions

The PANTHER classification system was used to analyse over-representation of GO terms for the list of genes that were located in significant genomic regions for all six traits, as well as for a shorter list of genes from regions that explained more than 3.0% of the genomic variance. Several significant biological process and molecular function GO terms were over-represented in the full list of genes (Table 6). The most significant molecular functions were “Type I interferon receptor binding” and “Cytokine activity”. Most of the 39 over-represented biological process terms were related to immune function, both innate and acquired. The most significant biological process identified was “Positive regulation of peptidyl-serine phosphorylation of STAT protein”. The signal transducer and activator of transcription (STAT) protein family regulates both type I and type II interferon receptors, immune function and cell proliferation. PANTHER analysis of genes located in regions associated with more than 3.0% of the genomic variance identified only two significantly over-represented molecular function terms (Table 7), “Glutathione transferase activity” and “Transferase activity, transferring alkyl or aryl (other than methyl) groups”.

**Table 6:** List of ontology terms that were significantly over- and underrepresented in the set of genes located in 1-Mb windows that were identified for at least one temperature-humidity index category comparison

Ontology term	Gene set				
	Annotated genes^a^ (n = 21,324)	Genes^b^ (n = 254)	Number of genes expected	Over (+) or under (−) represented	*p* value
Biological process					
Positive regulation of peptidyl-serine phosphorylation of STAT protein	36	10	0.43	+	2.37E−07
Natural killer cell activation involved in immune response	37	10	0.45	+	3.08E−07
Regulation of peptidyl-serine phosphorylation of STAT protein perception	38	10	0.46	+	3.98E−07
B cell proliferation	48	10	0.58	+	3.70E−06
Natural killer cell activation	50	10	0.60	+	5.45E−06
Response to exogenous dsRNA	51	10	0.61	+	6.58E−06
T cell activation involved in immune response	56	10	0.67	+	1.59E−05
Mononuclear cell proliferation	73	11	0.88	+	1.44E−05
Lymphocyte proliferation	73	11	0.88	+	1.44E−05
Leukocyte proliferation	77	11	0.93	+	2.49E−05
Response to dsRNA	72	10	0.87	+	1.66E−04
Positive regulation of peptidyl-serine phosphorylation	88	11	1.06	+	9.63E−05
Defense response to virus	110	13	1.33	+	9.08E−06
Lymphocyte activation involved in immune response	88	10	1.06	+	1.04E−03
B cell differentiation	89	10	1.07	+	1.16E−03
Regulation of peptidyl-serine phosphorylation	112	12	1.35	+	1.14E−04
Humoral immune response	96	10	1.16	+	2.29E−03
Leukocyte activation involved in immune response	111	12	1.34	+	8.36E−03
Cell activation involved in immune response	113	10	1.36	+	9.79E−03
Adaptive immune response	137	12	1.65	+	9.80E−04
T cell activation	161	14	1.94	+	9.81E−05
B cell activation	117	10	1.41	+	1.33E−02
Response to virus	158	13	1.90	+	6.05E−04
Regulation of STAT cascade	125	10	1.51	+	2.37E−02
Regulation of JAK-STAT cascade	125	10	1.51	+	2.37E−02
Lymphocyte differentiation	171	12	2.06	+	9.79E−03
Lymphocyte activation	241	15	2.90	+	2.17E−03
Leukocyte differentiation	230	14	2.77	+	6.96E−03
Cell proliferation	322	19	3.88	+	1.33E−04
Immune effector process	257	15	3.10	+	4.81E−03
Response to organic cyclic compound	377	20	4.54	+	3.13E−04
Defense response to other organism	264	14	3.18	+	3.34E−02
Leukocyte activation	284	15	3.42	+	1.62E−02
Cell activation	357	18	4.30	+	3.08E−03
Response to nitrogen compound	471	21	5.68	+	2.47E−03
Response to organic substance	1460	43	17.60	+	3.57E−04
Cell differentiation	2021	49	24.36	+	1.22E−02
Cellular developmental process	2077	49	25.03	+	2.59E−02
Developmental process	3175	67	38.27	+	1.45E−02
Sensory perception	1326	1	15.98	–	7.80E−03
Molecular function					
Type I interferon receptor binding	32	10	0.39	+	2.61E−08
Glutathione transferase activity	27	5	0.33	+	4.88E−02
Cytokine activity	177	17	2.13	+	2.12E−07
Cytokine receptor binding	218	15	2.63	+	2.12E−04
Receptor binding	1030	33	12.41	+	8.36E−04
G-protein coupled receptor activity	1445	3	17.42	–	4.06E−02
Cellular component					
Cytoplasm	7080	118	85.33	+	1.70E−02

^a^Number of genes in the *Sus scrofa* 10.2 annotation set with given GO term. Total number of annotated genes in the *Sus scrofa* 10.2 annotation is in parentheses

^b^Number of genes with given GO term located in genomic regions explaining at least 1% of genomic variance for one of more THI comparison GWAS. Total number of annotated genes residing in the selected regions is in parentheses

**Table 7:** List of ontology terms that were significantly over- and underrepresented in the set of genes located in 1-Mb windows that were associated with more than 3% of genetic variance for at least one temperature-humidity index category comparisons

Ontology term	Gene set				
	Annotated genes^a^ (n = 21,324)	Genes^b^ (n = 44)	Number of genes expected	Over (+) or under (−) represented	*p* value
Biological process					
None					
Molecular function					
Glutathione transferase activity	27	5	0.07	+	3.35E−05
Transferase activity, transferring alkyl or aryl groups	54	5	0.15	+	1.01E−03
Cellular component					
None					

^a^Number of genes in the *Sus scrofa* 10.2 annotation set with given GO term. Total number of annotated genes in the *Sus scrofa* 10.2 annotation is in parentheses

^b^Number of genes with given GO term located in genomic regions explaining at least 3% of genomic variance for one of more THI comparison GWAS. Total number of annotated genes residing in the selected regions is in parentheses

## Discussion

**Fig. 1:**
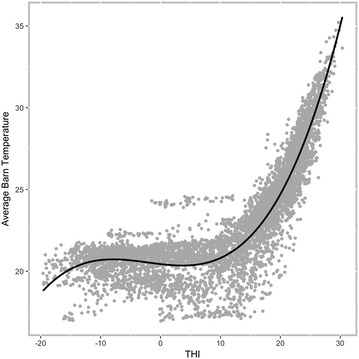
Temperature-humidity index (THI; °C) versus average barn temperature (°C) using a 3rd degree polynomial regression across all time periods when both measures were available

Environmental temperature affects feeding behaviour of pigs. In this study, THI was computed using outdoor temperature, but ideally, barn temperatures should be used. Although barn temperatures were collected using thermometers located at each end of the barn, for some groups of pigs there were numerous missing data points due to thermometer failure and other technical issues. Thus, THI from an on-site weather station was found to be a good predictor of barn temperature (adjusted R^2^ = 0.85; Fig. 1).

In this study, barrows from all three sire breeds had higher average daily RFID pings than gilts for each THI category. This is consistent with Brown-Brandl et al. [21], who reported that barrows spent more time at feeders than gilts. However, in a different study, Hyun et al. [22] reported no difference in time spent at feeders between sexes. In the study of Hyun et al. [22], the electronic feeding system allowed only one pig to eat at a time, while Brown-Brandl et al. [21] used an electronic feeding system like the one used here, consisting of one feeder with five feeding spaces. Current production systems use multi-space feeders since they are cost-effective and reduce negative social interactions during feeding. An interesting observation in the current study was that the difference in feeding activity between barrows and gilts increased with increasing temperatures for all sire breed by THI categories except Landrace sired pigs in the Emergency category. Thus, the observed difference in feeding activity between barrows and gilts may be due to competition for space or differences in how each sex handles heat stress.

We found that the impact of heat stress on feeding behaviour differs between breeds. Feeding activity of Duroc and Yorkshire sired pigs increased as THI increased, while that of Landrace sired pigs decreased as THI increased. Several approaches have been used to determine a pig’s ability to handle stressful situations. To test how a pig copes with a perceived stressful situation, the back test has been used, in which piglets are placed on their backs and time until first struggle or time spent struggling is recorded [23–26]. Time until first struggle is greater for animals that are calmer and that are capable of handling stressful situations better. Rohrer et al. [26] showed that time until first struggle during the back test is positively genetically correlated with number of meals per day and negatively genetically correlated with average meal length. As THI increased, Landrace sired pigs spent less time at the feeder, which suggests that the Landrace sired pigs were less able to handle stressful situations, heat stress in particular, or have a lower thermal comfort zone than Duroc and Yorkshire sired pigs. Hence, differences in heat tolerance can be observed through changes in feeding activity in grow-finish pigs when exposed to increased temperatures.

Several regions were identified in multiple THI category comparisons. One interesting comparison is how animals cope with the most extreme heat, i.e. Emergency THI. These comparisons had low estimates of heritability and typically detected few 1-Mb regions, which could reflect that all animals were considerably stressed during Emergency THI regardless of their genetic background. It should be noted that fewer animals experienced Emergency THI, so these analyses had fewer observations and a reduced power to detect associations. There were three regions (defined as chromosome_position in Mb) that were the same (SSC1_15, SSC12_12, and SSC14_11) for the traits including Emergency THI. Similar results should be expected as phenotypic correlations among these traits were relatively high, ranging from 0.80 to 0.94 (Table 1). The SSC14_11 region explained a large portion of the genomic variance in each of the analyses: 8.3, 9.8, and 4.2% for the comparison of the Emergency THI category to the Normal, Alert, and Danger THI categories, respectively. This region contains the *DPYSL2* gene, which is involved in the release of neural peptides from sensory neurons when stimulated [27]. A second possible candidate is the *ADRA1A* gene, which encodes an adrenergic receptor associated with response to stress hormones such as adrenaline and epinephrine [28]. The SSC12_12 region contains two genes that are associated with blood flow (*GNA13* and *AMZ2*), which may be interesting candidates for study. Once nerves detect an increase in heat, a signal is sent to the hypothalamus that causes warmth-sensitive neurons to trigger a heat-loss reflex by either vasoconstriction or behavioural mechanisms [29]. Moran et al. [30] postulated that one critical component to thermal tolerance is an individual’s ability to direct greater blood flow to the skin for heat dissipation. Unlike most other mammals, pigs have a limited capacity to use water evaporation to lose heat [31], so dissipation of heat through skin is critical to their thermal regulation.

A second interesting comparison is how animals change their behaviour when temperatures exceed Normal values. In these comparisons, estimates of heritability were moderate and more regions of interest were detected than in analyses comparing the three levels of heat stress (Alert, Danger and Emergency). Phenotypic correlations among these three traits were also high (range 0.68–0.88; Table 1) but not as high as the correlations with traits associated with Emergency THI. Six similar regions were identified in comparisons to the Normal category (SSC5_109, SSC6_15, SSC7_53, SSC10_44, SSC13_17 and SSCX_3). The SSC 7_53 region was detected in two of the three comparisons to Normal (Normal-Alert, and Normal-Danger) as well as the comparison of Alert-Danger and each association explained a relatively large amount of the genomic variance (4.8, 13.2 and 10.3%, respectively). Evaluation of this region identified a heat shock protein gene (*DNAJA4*), which is located at 53.2 Mb. Heat shock proteins protect cells from stressors [32]. The *DNAJA4* gene was shown to be expressed at higher levels after heat stress in chicken testes [33] and in several tissues in heat stressed rats [34] than in unstressed control animals. This region also contains members of the acetylcholine receptor subunit family. Two of these genes, *CHRNA3* and *CHRNB4*, form a complex that activates POMC neurons, which stimulate *MC4R* and regulate eating behaviour [35]. Thus, these three genes (*DNAJA4*, *CHRNA3*, and *CHRNA5*) warrant further investigation.

A potential candidate gene for the SSCX_3 region is *NLGN4X*, which is expressed in the brain. A mouse model in which this gene is knocked out showed that null mice had deficits in social interactions and communication with other mice [36]. An expanded region on SSC7 between 44 and 45 Mb, which was identified in the comparisons of Normal THI with the Alert and Danger categories, contains two heat shock protein genes, *HPS90AA1* and *HSP90AB1*, located at 45.1 Mb. The *HSP90AA1* gene encodes an inducible protein expressed during cellular stress that is more highly expressed in the testes of heat-stressed chickens [33] than in control birds. Polymorphisms in the *HSP90AA1* gene have been associated with adaptation to thermal conditions in sheep [37], while polymorphisms in the *HSP90AB1* gene have been associated with heat tolerance in cattle [38].

In order to gain a better biological insight into the genetic mechanisms that control heat tolerance in pigs, an enrichment analysis of gene function was performed using PANTHER. The most over-represented biological processes were related to the immune system. Several reports have also associated immune function genes with an animal’s response to heat stress. Moran et al. [30] summarized that an animal’s heat response cascade included three components beginning with heat shock proteins, followed by expression of interferon-inducible genes and concluding with small non-specific stress responses of specific cell lines. Islam et al. [39] found greater expression of inflammatory cytokines after exposure to heat stress in mice that were intolerant to heat relative to mice that were determined to be heat tolerant. Altered white blood cell counts and antibody production due to heat stress have also been documented [40] in poultry. Therefore, selection of immune function genes residing in the regions identified in this study warrant further investigation.

At the cellular level, heat stress disrupts normal folding of newly synthesized proteins [34], which then are not recognized as a native protein and will be targeted for degradation. The glutathione transferase pathway breaks down molecules, which are recognized as potential toxins or foreign material, and Stallings et al. [34] showed that genes in this pathway are upregulated during heat stress. We observed that the glutathione transferase molecular function was significantly over-represented in regions detected in both PANTHER analyses (Tables 6 and 7) confirming the importance of the glutathione transferase pathway as one of an animal’s biological mechanisms to cope with elevated temperatures.

## Conclusions

Changes in feeding activity are indicative of response to heat stress in grow-finish pigs. Individual differences in tolerance to heat have been identified in mice [39], man [30], and in pigs (current study) and our results show that thermal tolerance in pigs is heritable. Genes involved in immune response and function were among those over-represented in the regions associated with changes in feeding activity between different THI categories. Candidate genes identified in this work, including heat shock proteins and stress response, merit further investigation and may facilitate genetic selection for improved grow-finish performance during heat stress events. Selection for heat-tolerant grow-finish pigs would increase production efficiency.
